# MSI-1436 improves EMS adipose derived progenitor stem cells in the course of adipogenic differentiation through modulation of ER stress, apoptosis, and oxidative stress

**DOI:** 10.1186/s13287-020-02102-x

**Published:** 2021-02-03

**Authors:** Lynda Bourebaba, Katarzyna Kornicka-Garbowska, Mohamad Al Naem, Michael Röcken, Jacek Łyczko, Krzysztof Marycz

**Affiliations:** 1 Department of Experimental Biology, Wrocław University of Environmental and Life Sciences, Norwida 27B Street, A7 Building, 50-375 Wrocław, Poland; 2 International Institute of Translational Medicine, Malin, Jesionowa 11, 55-114 Wisznia Mała, Poland; 3 Faculty of Veterinary Medicine, Equine Clinic-Equine Surgery, Justus-Liebig-University, 35392 Giessen, Germany; 4 Department of Chemistry, Faculty of Biotechnology and Food Science, Wrocław University of Environmental and Life Sciences, Norwida 25, 50-375 Wrocław, Poland

**Keywords:** Adipogenesis, Equine metabolic syndrome, Insulin resistance, Trodusquemine

## Abstract

**Background:**

Protein tyrosine phosphatase 1B (PTP1B) is one of the major negative regulators of leptin and insulin signaling, and has been strongly implicated in insulin resistance development in the course of obesity and metabolic syndrome conditions; however, its exact role in controlling adipose tissue biogenesis is still poorly understood.

**Objectives:**

This investigation aimed to elucidate whether selective inhibition of PTP1B using MSI-1436 compound may improve and restore the defective adipogenicity of ASCs isolated from EMS-affected horses.

**Methods:**

Equine ASC EMS cells were cultured under adipogenic conditions in the presence of PTP1B inhibitor and were subsequently tested for expression of the main adipogenic-related genes using RT-qPCR, changes in free fatty acid profiles by means of GC-MS technique, and for mitochondrial dynamics improvement through the analysis of mitochondrial transmembrane potential and oxidative stress.

**Results:**

Selective inhibition of PTP1B in equine ASC EMS cells improved substantially adipogenic differentiation by promoting cellular proliferation and normalizing expression of *C/EBPalpha*, *PPARγ*, and *Adipoq* markers that are critical for proper adipogenesis. Levels of secreted adiponectin and *PPARγ* were also shown to be increased in MSI-1436-conditioned cells, while total leptin levels markedly dropped under the same conditions. Moreover, MSI-1436 treatment enabled the regulation of metabolic-related transcripts that are crosslink to adipogenesis, namely *Akt1*, *Akt2*, and *SHBG*. The obtained results demonstrated also an obvious reduction in intracellular accumulated ROS and NO, as well as mitigated ER stress through the downregulation of *Chop*, *Perk*, *Atf6*, *Ire1*, and *Xbp1* transcripts upon PTP1B inhibition. Furthermore, general fluctuations in FFA composition of all differentiated groups have been highlighted, where palmitic acid, palmitoleic acid, stearic acid, and linolelaidic acid that are known to be associated with the development of metabolic disorders were found to be normalized upon PTP1B inhibition during adipogenic differentiation.

**Conclusion:**

The presented data provides the evidence that the use of PTP1B inhibitor may be successful in controlling and enhancing adipogenic differentiation of impaired equine ASCs affected by metabolic syndrome, and thus offers new insights for the management of obesity through the regulation of adipose tissue dynamics.

**Graphical abstract:**

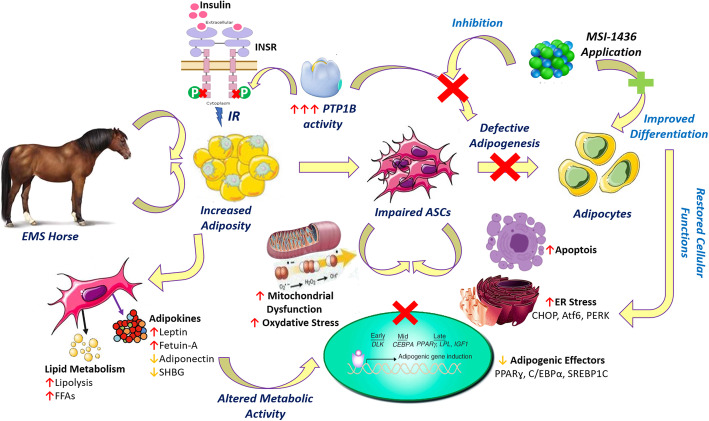

## Background

Metabolic syndrome in both human as well as in horses (defined as equine metabolic syndrome—EMS) has become more and more frequently diagnosed endocrine disorder [1, 2]. Its development is mainly caused by sedentary lifestyle, overfeeding with carbohydrates combined with lack of physical activity. As a consequence, affected individuals develop insulin resistance and related complications. In both species, metabolic syndrome culminates in the development of fatal cardiovascular diseases (in humans diabetic foot while in horses *laminitis*) [3]. Horses affected with EMS are characterized by deterioration of lipid metabolism and clinically specific lipid accumulation on the neck or on the base of tail and finally lipotoxicity, which is a key player in the insulin resistance development. Although obesity is not a critical component of EMS, since it was proved that lean horses also suffer for insulin resistance, involvement of adipose tissue deterioration in EMS and local inflammation seem to be indisputable [4–6].

Adipose tissue (AT) and adipose stem progenitor cells (ASCs) have been shown to be key factors in the development of obesity as well as insulin resistance [7–12]. AT is considered as an essential, active metabolic and hormonal organ that orchestrates many pathways involved in insulin resistance development. AT is responsible for the synthesis and secretion of several hormones including leptin, adiponectin, visfatin, or angiotenstin, which modulates insulin resistance and inflammatory axis [13]. Adipose tissue of EMS horses secretes a large amount of pro-inflammatory mediators including tumor necrosis factor α (TNF-α), interleukin-1 (IL-1), and interleukin-6 [4, 14].

Recently, sex hormone binding globulin (SHBG) and fetuin-A (AHSG) have been shown to play an important regulatory role in insulin resistance, and can thus represent promising targets for future therapeutics. Increased fetuin and deceased SHBG systemic levels are strongly associated with metabolic syndrome and type 2 diabetes development [15]. Growing evidence indicates that fetuin-A can modulate insulin-stimulated insulin receptor (INSR) and insulin receptor substrate 1 (IRS1) phosphorylation through the direct interaction with the insulin receptor β-subunit; moreover, fetuin-A is believed to strongly promote lipid-induced insulin resistance over the enhancement of free fatty acids binding to toll-like receptor 4 (TLR4). Furthermore, fetuin-A has been implicated in the pathophysiological mechanisms that lead to obesity onset; indeed, this protein has been claimed to mediate the reduction of adiponectin synthesis through the modulation of the Wnt3a/PPARγ pathway, and has been proposed as a chemoattractant for macrophage recruitment and migration into adipose tissue, promoting their subsequent polarization to M1 pro-inflammatory subtype, that contributes mainly to the AT inflammation [16]. To another extent, decreased levels of SHBG have been attributed to high levels of circulating insulin and glucose, as well as to monosaccharide-induced lipogenesis in the course of obesity and hyperinsulinemia, that may contribute to the repression of the gene that controls the production of SHBG [17]. Secretion of particular hormones or cytokines in AT in the course of EMS development partially results from the molecular deterioration of adipose stem progenitor cells that reside within. Recent data clearly indicates that ASCs as progenitor cells that give a rise to mature adipocytes play a critical role in the impairment of AT during metabolic syndrome development in both human and horses [18, 19]. Our and other groups’ early findings showed that accumulation of reactive oxygen species (ROS) combined with elevated inflammation is key components that impair adipogenic differentiation potential of ASCs and limit their multipotency [20, 21]. Deterioration of mitochondrial biogenesis and dynamics, and production and secretion of the so-called mitoproteins that are involved in adipocyte maturation are the major mechanisms which underlay that phenomenon. The ASC-derived mitochondria are characterized by membrane raptures, disarrayed cristae, and vacuole formation, which in turn contributes to the development of senescent phenotype and decreased proliferative potential. Furthermore, EMS condition seriously limits ASC viability as well as induces apoptosis of those cells and modulates hypertrophy of mature adipocytes [22–24]. Adipose stem progenitor cells under metabolic syndrome condition might be also partially involved in adipose tissue fibrosis, which is an important component of metabolic disorders. An excessive accumulation of oxidative stress factors, lipotoxicity, and inflammation strongly contribute to ASCs’ molecular impairment and in turn to the insulin resistance development [25]. Accumulation of free fatty acids (FFAs) in the blood of patients suffering from metabolic syndrome indicates on the correlation between obesity-related insulin resistance and inflammation; however, as mentioned above, not every horse which suffers from EMS is obese [5, 26]. Therefore, probably some other yet not discovered mechanisms may be involved in the development of adipose tissue insulin resistance. Several researches have underlined the importance of endoplasmic reticulum (ER) stress in the course of insulin resistance and inflammation development, since ER is a major site for the proteins, lipids, and sterol synthesis, which modulate adipocyte metabolism [27, 28]. The deterioration of ER functionality leads to increased accumulation of misfolded or unfolded peptides triggering the state of ER stress. Among proteins involved in the ER stress, inositol-requiring enzyme (IRE)-1, PKR-like ER-regulating kinase (PERK), and activating transcription factor (ATF)-6 play the key role [29, 30]. These proteins trigger the activation of pathways that mitigate ER stress, through the unfolded protein response (UPR). As a consequence, the production of protein chaperones needed for proper protein folding or by slowing protein synthesis is triggered; however, if unsuccessful, the unfolded proteins are removed from cells.

ER stress is also involved in the activation of several serine/threonine kinases, including c-jun NH2-terminal kinase (JNK) and IκB-α kinase (IKK). Moreover, it was shown that formation of the IRE-1α-TRAF 2 complex results in the activation and nuclear translocation of nuclear factor κB, which is a key promoter of inflammation. In turn, IRE-1α-TRAF 2 complex also activates JNK, which modulates the expression of pro-inflammatory cytokines and induces insulin resistance through serine phosphorylation of insulin receptor substrates 1 and 2 [27]. Little is known about the involvement of ER stress in the protein tyrosine phosphates’ (PTPs) activation, which are known to inactivate insulin receptor substrate through its dephosphorylation. Recently, MSI-1436 has been shown to inhibit PTP1B—a key enzyme regulating insulin and leptin signaling, via a non-competitive allosteric mechanism, which improves insulin sensitivity and reduces obesity. MSI-1436 is a specific, reversible, and non-competitive inhibitor of PTP1B, which targets preferentially the long form of PTP1B (1–405), containing the extended C-terminal segment [31]. It was shown that MSI-1436 is a potential anti-diabetic agent that can improve glucose tolerance, improve insulin sensitivity, and increase weight loss in insulin-resistant mice [31]. Therefore, the major goal of the presented investigation was to characterize the effects of MSI-1436 and thus PTP1B inhibition on adipogenic differentiation potential of ASCs isolated from EMS horses, with the special attention to ER stress, SHBG, and fetuin axis in order to define its potent involvement in the modulation of adipose tissue insulin resistance.

## Materials and methods

All chemicals and reagents were obtained from Sigma-Aldrich (Poznań, Poland), unless otherwise stated. Cell culture reagents were purchased from BioWest (VWR International, Gdańsk, Poland).

### Equine ASC isolation and cell culture

Animals from whose adipose tissue samples were collected were diagnosed in accordance with previously established protocol [20]. Clinical and biochemical parameters of these animals were described previously [20]. Subcutaneous adipose tissue biopsies were sampled from the tail base area of adult healthy and EMS horses, under local anesthesia induced by 2% lidocaine (Polfa S.A., Warsaw, Poland). Samples were immediately rinsed using Hanks’ Balanced Salt Solution (HBSS) supplemented with 1% antibiotics for the minimization of possible microbial contamination. Tissue samples were afterwards finely diced using sterile scalpel-blade, subjected to collagenase type I digestion (0.1 mg/mL) during 40 min at 37 °C and 5% CO_2_, and centrifuged at 1200×*g* for 10 min. The obtained cell pellet was resuspended in Dulbecco’s modified Eagle’s medium (DMEM) containing 1000 mg/L glucose, and supplemented with 5% of fetal bovine serum (FBS) and 1% of a penicillin and streptomycin (PS) solution in culture flasks. Cultures were maintained in a humidified CO_2_ incubator (37 °C, 5% CO_2_, 95% air atmosphere), passaged every 3 days (80–90% of confluence) using an Accutase® solution, and cells were used at third passage for experiments [32].

Cellular population purity was evaluated by means of fluorescence-activated cell sorting technique (BD FACSCalibur, Becton Dickinson, Franklin Lakes, NJ, USA). ASC phenotyping was assessed by flow cytometry analysis using fluorochrome-conjugated monoclonal antibodies (anti-CD105, Acris, Herford, Germany, SM1177PT; anti-CD45, Novus Biologicals, Littleton, CO, USA, NB1006590APC; anti-CD44, R&D Systems, Minneapolis, MN, USA, MAB5449; anti-CD90, ab225, Abcam, Cambridge, UK). Multipotency of isolated ASCs was evaluated through osteogenic, chondrogenic, and adipogenic differentiation of cells cultured in StemXVivo kits (R&D System). All abovementioned techniques were thoroughly described previously [9, 21, 33].

Isolated ASCs expressed significant amounts of positive cell surface markers, namely CD90 and CD105, and were negative for CD45 and CD34, which excluded their hematopoietic origin. Moreover, multipotency of ASCs was confirmed by efficient differentiation into osteoblast, chondrocytes, or adipocytes in vitro [9, 20, 21].

### Adipogenic differentiation of equine ASCs

To investigate the potential effect of PTP1B inhibition using MSI-1436 on adipogenic differentiation efficiency, ASCs derived from either healthy (HE) or EMS horses were firstly seeded onto culture plates in regular DMEM culture medium and cultured until they reached confluence. Adipogenic differentiation was then initiated using the StemPro™ Adipogenesis Differentiation Kit (Gibco™, Thermo Fisher Scientific, Poland), following the manufacturer’s instruction. All groups of cells were cultured in adipogenic medium for 14 days in the presence or absence of MSI-1436 inhibitor at a concentration of 1 μM, and culture media were changed every 3 days.

In parallel, HE and EMS cells incubated in the basal medium including low glucose DMEM and 5% FBS and 1% P/S were used as negative controls for both phenotypes.

At the 15th day of differentiation process, all cultures were stopped and subjected to further analysis described below.

### Bromodeoxyuridine incorporation assay

DNA synthesis and cell proliferation extend were assessed using the 5-bromo-2-deoxyuridine (BrdU) Cell Proliferation ELISA Kit (Abcam, Cambridge, UK) according to the manufacturer’s recommendations. Briefly, adipogenic and undifferentiated ASCs were incubated with BrdU reagent 24 h prior to differentiation arrest, and left overnight at 37 °C. Incorporation of BrdU into cellular DNA was determined by labeling fixed cells with anti-BrdU monoclonal antibody, and goat anti-mouse IgG conjugated with horseradish peroxidase (HRP) as a secondary antibody. HRP substrate degradation degree was measured with a spectrophotometer plate reader (Spectrostar Nano; BMG Labtech, Ortenberg, Germany) at a wavelength of 450 nm [34].

### Oil red O staining

The intracellular accumulation of neutral lipids within the differentiated and undifferentiated ASCs was evaluated by Oil Red O staining according to supplier’s instructions. Briefly, the cells were fixed in 4% paraformaldehyde for 40 min at room temperature, and additionally for 5 min with 60% isopropanol. The fixed cells were subsequently stained with 0.5 g/mL Oil Red prepared in 60% aqueous isopropanol for 15 min at room temperature, and then washed with 60% aqueous isopropanol and PBS. The nuclei were counterstained with hematoxylin solution for 1 min. All stained cells were monitored under an inverted microscope (Axio Observer A1, Zeiss), and micrographs were acquired using a Canon PowerShot digital camera.

### Detection of intracellular lipid droplets

The accumulation of neutral lipid droplets in adipogenic ASCs was visualized using the Oil Red O dye as described previously [35]. Briefly, ASCs were fixed with 4% paraformaldehyde for 40 min at room temperature at the 15th day of differentiation induction, followed by incubation with 60% isopropanol for 5 min. Slides were then incubated with Oil Red O for 15 min. The cells were also counterstained with hematoxylin for 1 min. Excessive dye was washed away with PBS. Photos were acquired using Axio Observer A1 inverted microscope (Zeiss, Oberkochen, Germany), while the photographic documentation was made using Canon PowerShot digital camera (Ota, Tokio, Japan).

### Analysis of viability and cell apoptosis using flow cytometry

Adipogenic EMS and healthy ASC apoptosis, dead cells, and viability were assessed using the multifunctional Muse Annexin V & Dead Cell Assay kit™ (Cat. No. MCH100105, Merck Millipore, Darmstadt, Germany), according to the manufacturer’s protocol. All differentiated-treated and untreated cells were collected, washed with HBSS, and labeled with the Annexin V & Dead Cell kit for 20 min at room temperature. The distribution of cells across the four populations (i.e., (i) non-apoptotic cells, not undergoing detectable apoptosis, Annexin V (−) and 7-AAD (−); (ii) early apoptotic cells, Annexin V (+) and 7-AAD (−); (iii) late apoptotic cells, Annexin V (+) and 7-AAD (+); and (iv) cells that have died through non-apoptotic pathway, Annexin V (−) and 7-AAD (+)) was determined by the use of Muse Cell Analyzer (Merck Millipore, Darmstadt, Germany).

### Multicaspase activity detection

The activity of caspase 1, 3, 4, 5, 6, 7, 8, and 9 was monitored using a Muse MultiCaspase assay kit (Cat. No. MCH100109, Merck Millipore, Darmstadt, Germany) following the user’s guide. All experimental groups of cells were mixed with 5 μL of Muse™ multicaspase reagent working solution and incubated for 30 min in a 37 °C incubator. Thereafter, 150 μL of Muse™ caspase 7-aminoactinomycin D (7-AAD) working solution was added to each sample and incubated in the dark for 5 min at room temperature. Multicaspase activity was subsequently assessed using a Muse Cell Analyzer (Merck Millipore, Darmstadt, Germany).

### Mitochondrial membrane potential detection assay

Changes in the mitochondrial membrane potential (MMP) were analyzed by measuring the incorporated MitoPotential lipophilic cationic dye, using the Muse™ MitoPotential Assay kit (Cat. No. MCH100110, Merck Millipore, Darmstadt, Germany). Following adipogenic differentiation in the presence or absence of MSI-1436 inhibitor, EMS and healthy cells were washed with HBSS and stained with the provided fluorescent dyes for 30 min at 37 °C, and the percentage of total depolarized cells (depolarized live + depolarized dead) was evaluated by the mean of a Muse Cell Analyzer (Merck Millipore, Darmstadt, Germany).

### Intracellular reactive oxygen and nitrogen species determination

Quantitative measurements of intracellular ROS and RNS, namely superoxide and nitric oxide radicals, were performed using the flow cytometry-based Muse® Oxidative Stress kit and Muse® Nitric Oxide kit (Cat. No. MCH100111/MCH100112, Merck Millipore, Darmstadt, Germany) respectively according to the users’ guide instructions. Cells from each treatment were collected at the 15th day of differentiation, washed with HBSS, and then resuspended in Muse Oxidative Stress and/or Muse Nitric Oxide Reagent working solutions for 30 min at 37 °C in the dark. Determination of ROS^+^/NO^+^ versus ROS^−^/NO^−^ populations was achieved using a Muse Cell Analyzer (Merck Millipore, Darmstadt, Germany).

### Measurement of adiponectin, leptin, and PPARγ levels in cultured media

Secretion of adiponectin, leptin, and PPARγ into the medium of the differentiated and undifferentiated ASCs was evaluated using commercial ELISA kits, as recommended by the manufacturer. In brief, cell-free supernatants were collected following 14 days of adipogenesis in the presence or not of PTP1B inhibitor, as well as from undifferentiated control HE and EMS cells. All conditioned culture media were then placed on ready-to-use microwell plates coated with antibodies against adiponectin, leptin, and PPARγ, respectively (Cat. No. MBS081354/MBS936352/MBS016871, MyBioSource, San Diego, USA), incubated with a biotin-labeled secondary antibody and a streptavidin horseradish-peroxidase (HRP) conjugate. The colorimetric reaction was initiated by addition of an HRP substrate (tetramethylbenzidine/peroxide), and measured at 450 nm in a spectrophotometer plate reader (Spectrostar Nano; BMG Labtech, Ortenberg, Germany). Adiponectin, leptin, and PPARγ concentrations were calculated based on the respective dose-response calibration curves that were built using horse adiponectin, leptin, and PPARγ standards provided in the ELISA kits.

### RNA preparation and quantitative RT-PCR for gene expression analysis

Total RNA of MSI-1436-treated adipogenic EMS cells as well as adipogenic and undifferentiated EMS and healthy untreated cells was collected using the TRIzol method according to the manufacturer’s protocol. RNA purity and concentration were established using a nanospectrophotometer (WPA, Biowave II, Germany). Genomic DNA (gDNA) digestion and cDNA synthesis were performed using a PrimeScript™ RT Reagent Kit with gDNA Eraser (TaKaRa, Gdańsk, Poland) by the mean of a T100 Thermal Cycler (Bio-Rad, Hercules, CA, USA) according to the manufacturer’s instructions.

Expression levels of targeted genes (Table 1) were analyzed by real-time reverse transcription polymerase chain reaction (RT-PCR), using a SensiFAST SYBR Green Kit (Bioline, London, UK) in a CFX Connect™ Real-Time PCR Detection System (Bio-Rad). Reactions performed in a 10-μL volume containing 150 ng cDNA were subjected to the following cycling conditions: 95 °C for 2 min, followed by 40 cycles at 95 °C for 15 s, annealing for 15 s, and elongation at 72 °C for 15 s. All results were normalized to glyceraldehyde 3-phosphate dehydrogenase (GAPDH) expression. The relative expression level was calculated by comparison of the tested groups with control group using the 2^-ΔΔCQ^ method [36].

**Table 1:** Sequences of primers used in qPCR

Gene	Primer	Sequence 5′–3′	Amplicon length (bp)	Accession no.
***PPARγ***	F:	TCCCTGTTTGTGTACAGCCTT	191	XM_014846252.1
	R:	CTCCATGGCTGATTTCCCCT		
***ADIPOQ***	F:	GGAGATCCAGGTCTTGTTGG	162	XM_014843352.1
	R:	TCGGGTCTCCAATCCTACAC		
***Lep***	F:	CACACGCAGTCAGTCTCCTC	176	XM_014854289.1
	R:	CGGAGGTTCTCCAGGTCAT		
***CEBPA***	F:	TCCCGGAGGGACCAAAGTTA	116	XM_023649498.1
	R:	CTCACATTGCACAAGGCACC		
***AKT1***	F:	CCAGGCTTGTGGTTGTCATCCT	108	XM_023628568.1
	R:	TTCTTGAGGAGGAAGTACCGGG		
***AKT2***	F:	CAGGAAACACAGGGAGCGG	160	XM_023649744.1
	R:	GACACGCTGTCACCTAGCTC		
***SHBG***	F:	GGCAACCTTTAACGCTCCAC	274	XM_023653464.1
	R:	GACAGGCTTTTGTCCTGGGT		
***AHSG***	F:	TGATGACCCCGAAACAGAGC	80	XM_005601872.3
	R:	CGTGCTTGTAGCCCTGATGA		
***p53***	F:	TACTCCCCTGCCCTCAACAA	252	U37120.1
	R:	AGGAATCAGGGCCTTGAGGA		
***Bax***	F:	TTCCGACGGCAACTTCAACT	204	XM_005596728.1
	R:	GGTGACCCAAAGTCGGAGAG		
***Bcl-2***	F:	TTCTTTGAGTTCGGTGGGGT	164	XM_014843802.1
	R:	GGGCCGTACAGTTCCACAA		
***p21***	F:	GAAGAGAAACCCCCAGCTCC	241	XM_003365840.2
	R:	TGACTGCATCAAACCCCACA		
***Casp-3***	F:	GGCAGACTTCCTGTATGCGT	167	XM_023630401.1
	R:	CCATGGCTACCTTGCGGTTA		
***Casp-9***	F:	TCCTACTCCACCTTCCCAGG	150	XM_005607504.3
	R:	CTCCGAAACAGCGTGAGCTA		
***PERK***	F:	GTGACTGCAATGGACCAGGA	283	XM_014852775.1
	R:	TCACGTGCTCACGAGGATATT		
***CHOP***	F:	AGCCAAAATCAGAGCCGGAA	272	XM_014844003.1
	R:	GGGGTCAAGAGTGGTGAAGG		
***IRE1***	F:	GAATCAGACGAGCACCCGAA	300	XM_023652216.1
	R:	TTTCTTGCAGAGGCCGAAGT		
***ATF6***	F:	CAGGGTGCACTAGAACAGGG	161	XM_023640315.1
	R:	AATGTGTCTCCCCTTCTGCG		
***XBP1***	F:	CGATCGAGTACTGTTGCCCT	299	XM_014742035.2
	R:	GACGTTTGTCCAGTGACCCT		
***SOD1 (Cu/Zn SOD)***	F:	CATTCCATCATTGGCCGCAC	130	NW_001867397.1
	R:	GAGCGATCCCAATCACACCA		
***SOD2 (Mn SOD)***	F:	GGACAAACCTGAGCCCCAAT	125	NW_001867408.1
	R:	TTGGACACCAGCCGATACAG		
***CAT***	F:	ACCAAGGTTTGGCCTCACAA	112	XM_014851065.1
	R:	TTGGGTCAAAGGCCAACTGT		
***GAPDH***	F:	GATGCCCCAATGTTTGTGA	250	NM_001163856.1
	R:	AAGCAGGGATGATGTTCTGG		

Sequences: amplicon length and access numbers of the primer sets. *PPARγ* peroxisome proliferator-activated receptor gamma, *ADIPOQ* adiponectin, *Lep* leptin, *CEBPA* CCAAT/enhancer-binding protein alpha, *AKT1* serine-threonine protein kinase 1, *AKT2* serine-threonine protein kinase 2, *SHBG* sex hormone binding globulin, *AHSG* alpha 2-HS glycoprotein, *p53* tumor suppressor p53, *Bcl-2* B cell lymphoma 2, *Bax* BCl-2 associated X protein, *p21* cyclin-dependent kinase inhibitor 1, *Casp-3* caspase 3, *Casp-9* caspase 9, *PERK* PRKR-like endoplasmic reticulum kinase, *CHOP* C/EBP homologous protein, *ATF6* activating transcription factor 6, *IRE1* inositol-requiring enzyme, *XBP1* X-box binding protein 1, *SOD1 (Cu/Zn SOD)* copper-zinc-dependant superoxide dismutase (CuZnSOD), *SOD2 (Mn SOD)* manganese-dependent superoxide dismutase (MnSOD), *CAT* catalase, *GADPH* glyceraldehyde 3-phosphate dehydrogenase

### Lipid extraction and GC-MS-based lipidomics analysis

#### Cellular metabolite isolation

Total metabolites were extracted from equine healthy and EMS ASCs cultured under either basal or adipogenic conditions according to the procedure described previously [29]. Briefly, cells from all tested groups were collected following 14 days of differentiation, washed three times with cold HBSS, and suspended in an extracting mixture comprising chloroform/methanol/water mixture in a ratio of 20:50:20. Thereafter, suspended cells were subjected to ultra-sonication at room temperature in a water bath sonicator for 2 h and vortexed for 2 min. All samples were transferred to 1.5 mL centrifugation tubes and centrifuged at 4 °C, 18,000×*g* for 20 min. Afterwards, the obtained supernatants were collected for each sample and dried completely overnight and subjected to gas chromatography-mass spectrometry (GC-MS) analysis.

#### Gas chromatography-mass spectrometry fatty acid characterization

The global analysis of adipogenic cells’ free fatty acid profile and quantity was performed according to Bourebaba et al.’s [29] protocol. Briefly, crude lipid extract of adipogenic cells, with 30 μg of heptadecanoic acid methyl ester (Me. C17:0) (Sigma-Aldrich, Steinheim, Germany) as internal standard, was subjected to alkaline hydrolysis with 1 M potassium hydroxide solution in methanol. Thereafter, the solution was extracted with hexane and dried over anhydrous magnesium(II) sulfate. Then, after solvent evaporation, methylation of extracted free fatty acids with 14% solution of boron trifluoride in methanol was carried out. Obtained fatty acid methyl esters were extracted with hexane, washed with saturated sodium chloride solution, and dried over anhydrous magnesium(II) sulfate. Finally, the solution of fatty acid methyl esters was densified under nitrogen (up to 200 μL), placed into vial with glass insert, and subjected to GC/MS analysis.

Shimadzu GCMS-QP2020 (Kyoto, Japan) equipped with a ZB-FAME (Phenomenex, Torrance, CA, USA) column (60 m × 0.25 mm i.d. × 0.25 μm film thickness) was used for GC/MS analysis. GC operational conditions are as follows: injection temperature 280 °C; 80 °C kept for 2 min, then to 180 °C at 3.0 °C/min, and then raised to 240 °C at 8.0 °C/min and kept for 4 min; carrier gas—helium with linear velocity 35.0 cm/s; and split ratio 1:10. MS operational conditions are as follows: ion source temperature 220 °C, interface temperature 250 °C, electron impact (EI) ionization at 70 eV, and scanning mode from 40 to 400 m/z.

Identification of fatty acid methyl esters was performed in comparison with analytical standard Supelco 37 component FAME Mix (Bellefonte, PA, USA) analysis carried out under the same conditions. The identification was confirmed by comparison of the experimentally obtained mass spectra with those available in Lipids GC/MS Shimadzu Library for GCMS-QP2020 single quadrupole (Chromaleont srl, Messina, Italy), and only compounds with similarity score ≥ 90% were considered as correct hits. Quantification was based on the peak area normalization.

### Statistics

Results were expressed as mean ± SD (*n* = 3). All statistical analyses and graphical representations were performed using GraphPad Prism (San Diego, CA, USA). Statistical differences were established with a one-way analysis of variance (ANOVA) followed by Bonferroni post hoc multiple comparison test, as indicated. All *p* values lower than 0.05 (*p* < 0.05) are summarized with one asterisk (*), those at *p* < 0.01 use two asterisks (**), and those at *p* < 0.001 have three asterisks (***).

## Results

### Evaluation of morphology, proliferation rate, and adipogenic differentiation

**Fig. 1:**
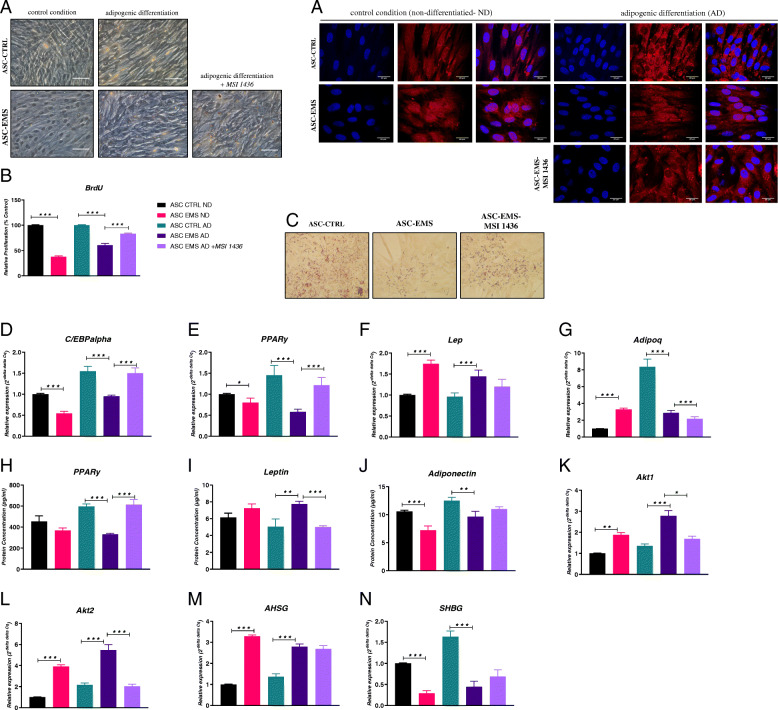
Evaluation of morphology, proliferation rate, and adipogenic differentiation of investigated cells. Visualization of cells cultured in standard growth medium (undifferentiated—ND) and cells which underwent adipogenic differentiation (AD) (**a**). BrdU assay for cell proliferation (**b**). Accumulation of lipid droplets was visualized with Oil Red O staining and cells were pictured with confocal microscope (**c**). Transcript levels of *C/EBPalpha* (**d**), *PPARγ* (**e**), *Lep* (**f**), and *Adipoq* (**g**) were determined with RT-qPCR. ELISAs revealed the extracellular levels of PPARγ (**h**), leptin (**i**), and adiponectin (**j**). Relative changes in the expression of genes related to adipogenesis and metabolic regulation: *Akt1* (**k**), *Akt2* (**l**), *AHSG* (**m**), and *SHBG* (**n**) established by RT-PCR. Results expressed as mean ± SD. *p < 0.05, **p < 0.01, ***p < 0.001. Magnification × 100; scale bar, 250 μm

Morphology of cells was investigated with brightfield microscope (Fig. 1a). In comparison to cells cultured in standard medium (undifferentiated), morphology of cells which underwent adipogenic differentiation changed from fibroblastic to spherical. Moreover, accumulation of lipid droplets in the cytoplasm was visible in differentiated cells. Analysis of BrdU incorporation revealed significant decrease in proliferation of ASC EMS ND in comparison to their healthy counterparts (Fig. 1b). Similar phenomenon was observed in the cells cultured in adipogenic differentiation medium. Interestingly, cells treated with PTP1B inhibitor, MSI-1436, displayed increased proliferation rate in comparison to untreated cells.

In order to visualize accumulation of lipid droplets, cells were stained with Oil Red O (Fig. 1c). Interestingly, ASC CTRL AD accumulated more lipid droplets in comparison to EMS counterparts while treating EMS cells with MSI-1436 inhibitor increased the accumulation of fatty acids. To support the staining results, RT-qPCRs for adipogenic factors were performed as well. We observed decreased expression of C/EBPalpha in both EMS groups (ND and AD) in comparison to healthy cells (ND and AD) (Fig. 1d). Interestingly, treating of EMS cells during adipogenesis with the PTP1B inhibitor significantly increased the expression of C/EBPalpha gene. The same phenomenon was observed for PPARγ expression (Fig. 1e). Interestingly, expression of Lep was elevated in EMS cells (ND and AD) and addition of the inhibitor did not influence its expression in cells (Fig. 1f). Expression of Adipoq was elevated in ASC EMS ND in comparison to their healthy counterparts (Fig. 1g). During adipogenic differentiation, mRNA levels of that gene were increased in CTRL cells in comparison to EMS while addition of the inhibitor reduced its expression. We have also investigated extracellular, secreted levels of proteins involved in adipogenesis progression using ELISAs. There were no differences in PPARγ amount in ND groups (Fig. 1h). Its levels were significantly decreased in ASC EMS AD in comparison to healthy cells while treating cells with MSI-1436 significantly increased its levels. In case of Lep, there were no differences between ND groups. Leptin amount was increased in EMS cells during adipogenic differentiation, and treating those cells with the inhibitor significantly reduced its levels (Fig. 1i). Adiponectin levels were decreased in EMS ND and AD cells in comparison to both CTRL ND and CTRL AD, respectively (Fig. 1j). Treating cells with the inhibitor did not influence adiponectin amount. Gene expression was evaluated with RT-qPCR. Akt1 expression was increased in EMS groups in comparison to healthy cells (Fig. 1k). Treatment of EMS cells with MSI-1436 significantly reduced Akt1 expression. The same phenomenon was noted for Akt2 expression (Fig. 1l). Its mRNA levels were upregulated in EMS cells; however, application of MSI-1436 reduced Akt2 expression. Expression of fetuin was increased in EMS cells in comparison to their healthy counterparts in both control and adipogenic conditions (Fig. 1m). Addition of the inhibitor did not affect AHSG expression. SHBG mRNA levels were diminished in EMS cells when comparing to control, and addition of PTP1B inhibitor did not influence significantly its expression levels (Fig. 1n).

### Evaluation of apoptosis

**Fig. 2:**
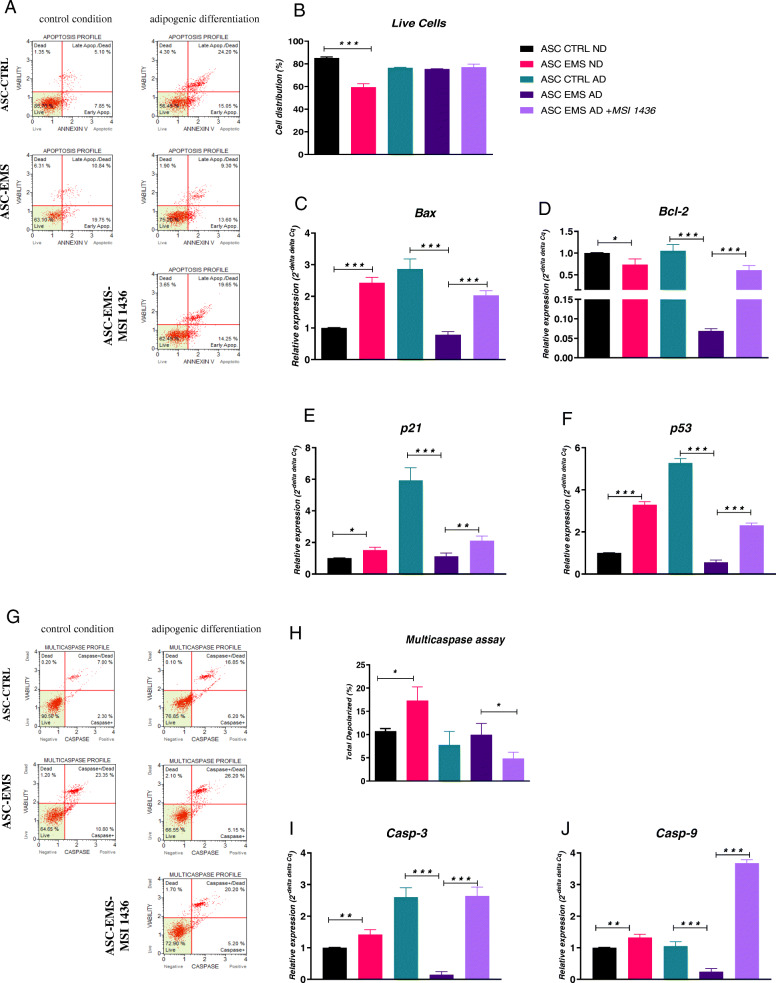
Evaluation of apoptosis. The Muse® Annexin V & Dead Cell assay (**a**, **b**) results showing increased apoptosis in EMS ND cells. Data was supported by the analysis of genes involved in the regulation of apoptotic pathway: *Bax* (**c**), *Bcl-2* (**d**), *p21* (**e**), and *p53* (**f**). The Muse® MultiCaspases assay results (**g**, **h**) indicate increased apoptosis in EMS cells. Furthermore, using RT-qPCR, expression of *Casp-3* (**i**) and *Casp-9* (**j**) was investigated. Results expressed as mean ± SD. *p < 0.05, **p < 0.01, ***p < 0.001

To determine the apoptotic profile of cells, we performed the Muse® Annexin V & Dead Cell assay (Fig. 2a, b). The number of live cells was decreased in EMS undifferentiated cells in comparison to their healthy counterparts, while no significant differences in viable cells were noted between the remaining experimental groups. We also performed RT-qPCR analysis of pro-apoptotic *Bax* gene expression (Fig. 2c). Interestingly, its expression was increased in EMS ND and decreased in EMS AD in comparison to both healthy cells (ND and AD). Treating cells with the inhibitor increased *Bax* expression. Anti-apoptotic *Bcl-2* mRNA levels were decreased in EMS ND in comparison to CTRL ND (Fig. 2d). During adipogenic differentiation, its expression was significantly reduced in EMS cells; however, addition of MSI-1436 inhibitor elevated its expression. The same phenomenon was noted for *p21* (Fig. 2e) and *p53* (Fig. 2f) expression. Their mRNA levels were reduced in EMS cells in comparison to the CTRL group; however, addition of the inhibitor increased their amount.

In order to estimate the caspase activation, we performed the Muse® MultiCaspases assay which allows for the quantitative measurement of caspase 1, 3, 4, 5, 6, 7, 8, and 9 simultaneously (Fig. 2g, h). We found that the number of caspase positive cells was increased in EMS ND in comparison to CTRL ND. Interestingly, no differences were noted between CTRL and EMS cells during adipogenic differentiation; however, addition of MSI-1436 reduced the number of dead cells in culture. Data from Muse® was further supported with RT-qPCR analysis of Casp-3 and Casp-9 expression. Interestingly, mRNA levels of Casp-3 were increased in EMS ND and decreased in EMS AD in comparison to the respective control groups (Fig. 2i). Furthermore, addition of the inhibitor significantly enhanced its expression. Interestingly, the same phenomenon was observed for Casp-9 expression (Fig. 2j).

### Evaluation of ER stress

**Fig. 3:**
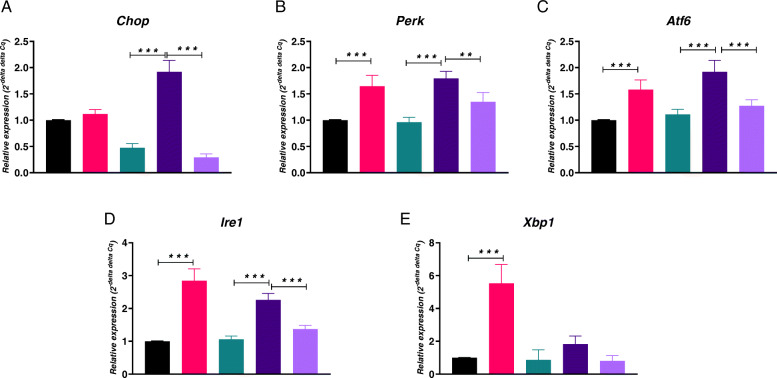
Expression of UPR-linked markers during adipogenesis and PTP1B inhibition. Transcript levels of *Chop* (**a**), *Perk* (**b**), *Atf6* (**c**), *Ire1* (**d**), and *Xbp1* (**e**). Results expressed as mean ± SD. *p < 0.05, **p < 0.01, ***p < 0.001

To determine the intensity of ER stress in cultures, we analyzed the mRNA levels of the UPR-related genes. Obtained results revealed significantly increased expression of *Chop* in EMS AD (Fig. 3a). However, PTP1B inhibition resulted in significantly diminished *Chop* mRNA levels. The expression of *Perk* was increased in EMS cells in both ND and AD conditions in comparison to CTRL cells (Fig. 3b). Addition of MSI-1436 ameliorated the expression of *Perk* in ASC EMS. The same phenomenon was observed for the expression of *Atf6* (Fig. 3c) and Ire1 (Fig. 3d) which indicates that PTP1B inhibition diminished ER stress in treated cells. ASC EMS ND were characterized by increased expression of *Xbp1* in comparison to control cells (Fig. 3e). Interestingly, there were no significant differences of *Xbp1* in adipogenic condition.

### Assessment of oxidative stress

**Fig. 4:**
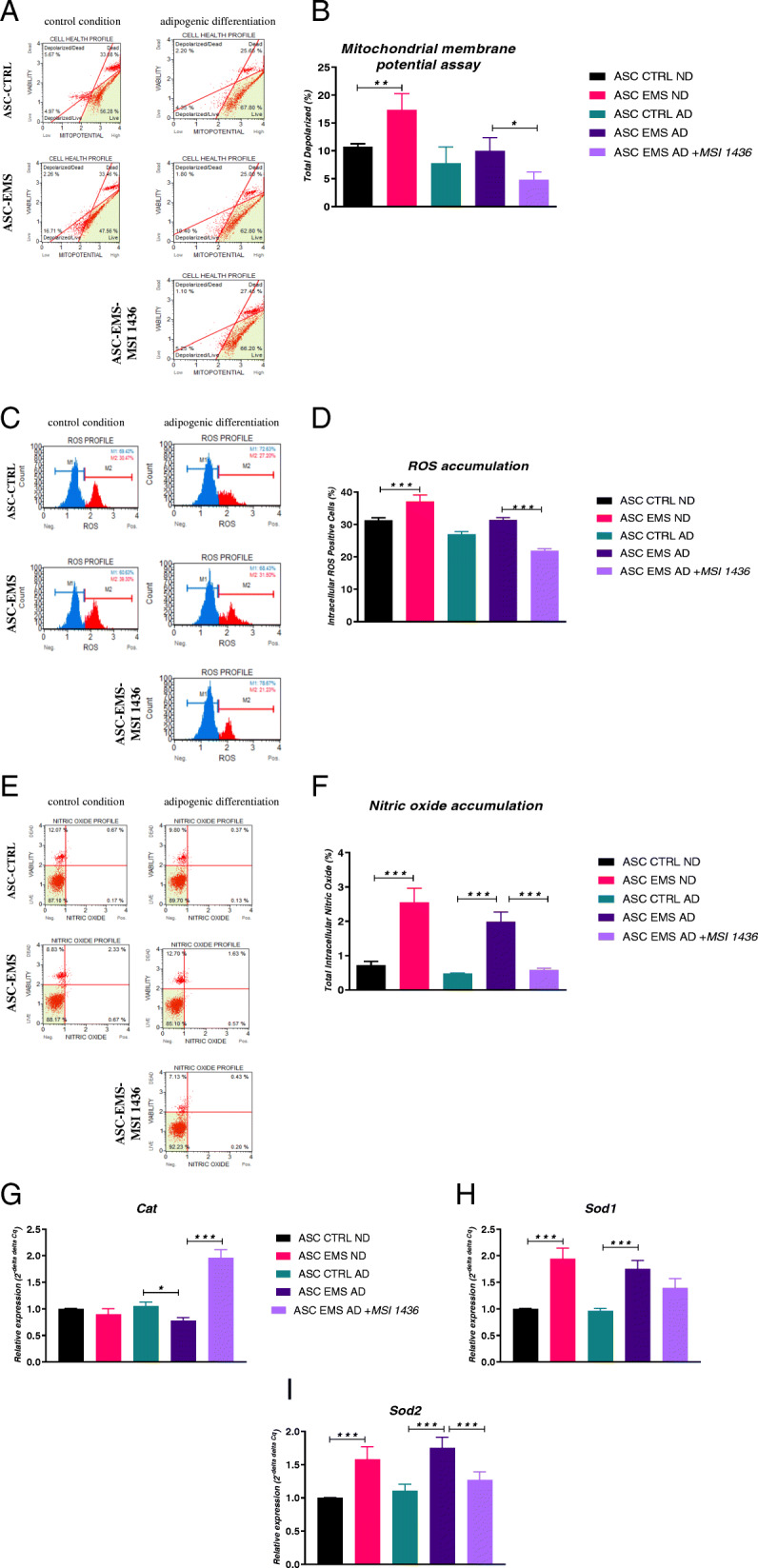
Assessment of oxidative stress in control and adipogenic conditions. Cells were subjected to flow cytometry-based system analysis and analyzed with Muse® MitoPotential kit (**a**, **b**), Muse® Oxidative Stress assay (**c**, **d**), and Muse® Nitric Oxide kit (**e**, **f**). Expression of antioxidative enzymes *Cat* (**g**), *Sod1* (**h**), and *Sod2* (**i**) was investigated with RT-qPCR. Results expressed as mean ± SD. *p < 0.05, **p < 0.01, ***p < 0.001

To assess the oxidative stress, cells were subjected to flow cytometry-based system analysis. Condition of mitochondria in cells cultured in standard condition and subjected to adipogenic differentiation was established with Muse® MitoPotential kit (Fig. 4a, b). Obtained data showed that the number of depolarized cells was increased in EMS ND in comparison to CTRL ND, while no differences between CTRL and EMS cells during adipogenic differentiation were noted. Interestingly, MSI-1436 inhibitor decreased the number of depolarized cells. Muse® Oxidative Stress assay (Fig. 4c, d) revealed increased accumulation of ROS in EMS ND in comparison to CTRL ND. Furthermore, in case of adipogenesis, treatment of EMS cells with PTP1B inhibitor significantly reduced ROS levels. Using the same machine, we investigated the accumulation of nitric oxide in cells with Muse® Nitric Oxide kit (Fig. 4e, f). Nitric oxide amount was increased in both EMS groups (ND and AD) in comparison to respective controls, while treatment of cells with MSI-1436 significantly diminished its levels. To support Muse findings, we performed RT-qPCR analysis to investigate the expression of antioxidative enzymes. No differences in *Cat* expression were observed between cells cultured in standard medium while decreased expression was found in EMS cells which underwent adipogenic differentiation in comparison to control (Fig. 4g). Interestingly, PTP1B inhibition significantly enhanced *Cat* expression in EMS cells during AD. Furthermore, we found that expression of *Sod1* was upregulated in EMS cells (ND and AD) in comparison to controls (Fig. 4h). Similar phenomenon was noted for *Sod2* expression; however, in that case, PTP1B inhibition resulted in significantly decreased *Sod2* mRNA levels (Fig. 4i).

### Evaluation of FFA metabolism

Lipid remodeling changes during adipogenesis of EqASCs were monitored using a GC-MS-based lipidomic profiling technique upon treatment with MSI-1436 inhibitor (Table 2). Overall, significant fluctuations in the levels of all detected fatty acids were observed in all differentiated groups as compared to undifferentiated cells. Among these fatty acids, myristic acid (C14:0), methyl pentadecanoate (C15:0), methyl elaidate (C18:1n, 9E), methyl octadecadienoate/linoleic acid (C18:2n, 9Z,11Z), and oleic acid (C18:1n) were significantly increased following adipogenic differentiation of healthy cells when compared to undifferentiated healthy cells (*p* < 0.01; *p* < 0.001). Moreover, decreasing levels of the same myristic acid (C14:0), methyl pentadecanoate (C15:0), methyl elaidate (C18:1n, 9E), and methyl octadecadienoate/linoleic acid (C18:2n, 9Z,11Z) were observed in EMS adipogenic cells in opposition to healthy ASCs differentiated cells (Table 2). By contrast, only myristic acid (C14:0) and methyl octadecadienoate/linoleic acid (C18:2n, 9Z,11Z) levels were maintained in EMS adipogenic MSI-1436-treated cells as compared to both healthy and EMS untreated adipogenic cells. To another extent, GC-MS profiling revealed a consequent decrease in both palmitic acid (C16:0), palmitoleic acid (C16:1), stearic acid (C18:0), and linolelaidic acid (C18:3N, 6Z, 9Z, 12Z) levels in healthy differentiated cells (*p* < 0.001). EMS differentiated cells exhibited lower amounts of palmitic acid (C16:0) and stearic acid (C18:0), and higher abundance of palmitoleic acid (C16:1) and linolelaic acid (C18:3N, 6Z, 9Z, 12Z) as compared to healthy group of cells (*p* < 0.001). Inhibition of PTP1B using MSI-1436 compound during adipogenesis induction enabled the normalization of the abovementioned fatty acids’ (palmitic acid, palmitoleic acid, stearic acid, and linolelaidic acid) concentrations in regard to EMS untreated cells (*p* < 0.001).

**Table 2:** Fatty acid composition of the FFA lipid class in equine ASCs healthy, EMS, adipogenic treated, and untreated cells

FFAs	Retention time (min)	Mass spectra match [%]	EqASCs_HE ND	EqASCs_EMS ND	EqASCs_HE AD	EqASCs_EMS AD	EqASCs_EMS+MSI-1436 AD
			Content (μg/2 × 10^**6**^ cells)				
*Tetradecanoic acid, methyl ester; ME. C14:0*	24.24	95	2.64 ± 0.04	4.89 ± 0.17^***^	3.15 ± 0.25^**;###^	2.745 ± 0.10^*;###^	3.24 ± 0.04^##^
*Pentadecanoic acid, methyl ester; ME. C15:0*	26.92	95	0.735 ± 0.10	0.21 ± 0.08^*^	0.81 ± 0.08^#^	1.05 ± 0.08^###^	1.8 ± 0.42^***;###^
*Hexadecanoic acid, methyl ester; ME. C16:0*	29.5	92	40.23 ± 0.59	68.325 ± 0.27^***^	28.47 ± 0.17^***;###^	24.96 ± 0.21^***;###^	30.195 ± 0.31^***;###^
*9-Hexadecenoic acid, methyl ester, (E)-; ME. C16:1N(9E)*	30.7	91	23.745 ± 0.02	3.165 ± 0.23^***^	2.235 ± 0.10^***;###^	4.725 ± 0.10^***;###^	2.19 ± 0.08^###^
*Octadecanoic acid, methyl ester; ME. C18:0*	34.22	96	36.75 ± 0.46	52.26 ± 0.17^***^	19.125 ± 1.5^***;###^	13.695 ± 0.10^***;###^	17.685 ± 0.10^**;###^
*9-Octadecenoic acid (Z)-, methyl ester; ME. C18:1N(9Z)*	34.8	93	1.275 ± 0.06	7.85 ± 0.27^***^	19.125 ± 1.5^***;###^	8.82 ± 0.04^***^	8.835 ± 0.06^***^
*9-Octadecenoic acid (E)-, methyl ester; ME. C18:1N(9E)*	34.9	92	2145 ± 0.148	17.085 ± 0.36^***^	5.115 ± 0.10^***;###^	2.145 ± 0.15^***;###^	1.545 ± 0.19^***;##^
*9,11-Octadecadienoic acid, methyl ester, (9Z,11Z); ME. C18:2N(9Z,11Z)*	36.6	90	1.185 ± 0.02	1.38 ± 0.04^***^	1.365 ± 0.02^**^	1.5 ± 0.04^***^	1.845 ± 0.02^***;###^
*6,9,12-Octadecadienoic acid, methyl ester (all Z); ME. C18:3N(6Z, 9Z, 12Z)*	34.29	94	21.225 ± 0.06	1.71 ± 0.25^***^	1.59 ± 0.08^***^	5.385 ± 0.06^***;###^	0.495 ± 0.14^***;###^

*Different from Healthy ASCs (*p < 0.05, **p < 0.01, ***p < 0.001)

^#^Different from EMS cells ASCs

## Discussion

In the presented study, for the first time, we have investigated the effects of MSI-1436 (trodusquemine) on the adipogenic differentiation of healthy and metabolically impaired mesenchymal stem cells. MSI is a selective, PTP1B inhibitor which protects rodents from diet-induced obesity (DIO) and for that reason is now being tested in clinical trials as an anti-diabetic agent for humans (in accordance with ClinicalTrials.gov, 4 studies have been performed). However, all of the molecular mechanisms of action and outcomes of MSI-1436 in different types of cells remain elusive. We have found that application of MSI-1436 restores adipogenic differentiation potential in EMS derived ASC, ameliorates ER stress, and modulates fatty acid composition of cells and mitochondrial bioenergetics. Our study provides novel evidence for the role of PTP1B in adipocyte dysfunction during obesity and underlines the role of its inhibitor in disease prevention and treatment.

In our previous studies, we have shown that adipogenic differentiation of ASC is altered in EMS animals, which correlates with adipose tissue hypertrophy and inflammation [20, 33]. Dysfunctional adipose tissue remodeling contributes to insulin resistance, accelerates fibrosis and inflammation, and secretes wide range of adipokines, which worsens metabolic state of the organisms [4]. MSI-1436 action has been mostly investigated with HepG2 cells in vitro and in DIO models in vivo [29]. It was shown to improve insulin-stimulated tyrosine phosphorylation of insulin receptor (IR) β and enhance insulin sensitivity, suppress appetite, reduce body weight, and decrease leptin and insulin plasma levels [31, 37]. Yet, little attention has been paid toward molecular mechanisms in adipocytes and changes in their metabolism after treatment. For that reason, to increase the knowledge regarding potential effects of MSI-1436 in the clinic, we decided to investigate its effects on adipogenic differentiation of ASC and their cytobiological properties.

Data from the present and previous studies indicates that deterioration of adipose tissue metabolism and plasticity contributes to the development of metabolic disorders [25]. As MSI-1436 was shown to protect against diabetes and enhance insulin sensitivity, we decided to unravel for the first time its effects on adipose tissue progenitor stem cells during control (standard culture medium, ND) and adipogenic (adipogenic differentiation medium, AD) conditions. We have found that EMS cells displayed decreased proliferation rate in both ND and AD conditions which stands with a good agreement with our and other studies, which showed that metabolically impaired cells suffer from decreased proliferation potential [18, 20, 21]. However, MSI-1436 treatment significantly increased growth kinetics of cells as we observed increased BrdU incorporation. Similarly, Smith et al. [37] revealed enhanced proliferation of zebra fish cardiomyocytes and mouse skeletal muscle satellite cells after MSI-1436 application. Obtained results clearly indicate that MSI-1436 promotes proliferation of metabolically impaired cells and restores progression of cell cycle. It is especially important since it was shown that hyperinsulinemia leads to cell cycle-induced senescence [38]. Adipogenic differentiation is a complex, multistep process in which preadipocyte differentiation is controlled and mediated by the set of signaling proteins and transcription factors. C/EBPalpha and PPARγ play a crucial role in the early stages of the process [39]. In the presented study, we have found that both of these genes’ mRNA levels were downregulated in EMS cells during ND and AD conditions. Similar phenomenon was observed in the study performed by Matulewicz et al. [40], who found decreased expression of *C/EBPalpha* and *PPARγ* in the subcutaneous adipose tissue samples of obese patients. Defects in PPARγ expression were shown to deteriorate adipose tissue function, plasticity, and lipotoxicity [41] which correlates with metabolic profile of EMS derived ASC [21]. On the other hand, activation of PPARγ signaling triggers crucial metabolic pathways in adipocytes and maintains their homeostasis. In obesity, PPARγ activation diminishes ectopic lipid accumulation, reduces inflammation, and improves insulin sensitivity and lipid metabolism. For that reason, targeting PPARγ activity in patients with type 2 diabetes and pre-diabetic insulin resistance is considered as a potential therapeutic strategy. Interestingly, in the presented study, we have found that PTP1B inhibition significantly increased *PPARγ* expression and protein levels which resulted in augmented adipogenic differentiation. It was shown that insulin resistance and inflammation reduce the ability of ASC to differentiate into mature adipocytes by suppressing PPARγ and insulin signaling pathway [42], leading to adipocyte hypertrophy in order to meet the demand for increased triglyceride storage. Furthermore, Bakker et al. have shown that preadipocyte number is significantly reduced in obese subjects [43]. Impaired adipose tissue turnover and disbalance between adipocytes’ death and maturation exaggerate disease progression. For that reason, restoration of adipose tissue plasticity by triggering progenitor stem cell differentiation is considered as an anti-diabetic strategy. Interestingly, in the presented study, we have found that PTP1B inhibition in progenitor cells increased PPARγ activity and enhanced adipogenic differentiation indicating a great therapeutic role of MSI-1436 in the restoration of adipose tissue plasticity. Our findings appear to be consistent with previous investigations performed by Song et al. [44] who showed that PTP1B negatively regulates and impairs adipocyte differentiation in obesity through TNFα. What is more, PTP1B was shown to negatively regulate adipogenesis of brown adipocytes [45]. Another evidence of PTP1B role in adipogenesis comes from the study performed by Owen et al. [46], who revealed that adipocyte-specific PTP1B depletion enhances lipogenesis and regulates glucose homeostasis. Adipose tissue modulates the functions of multiple other tissues in the body through the secretion of wide range of cytokines, the so-called adipokines. Among them, leptin and adiponectin are recognized as key players in the development of insulin resistance, obesity, and diabetes. It was shown that low levels of adiponectin, increased concentration of leptin, and leptin resistance correlate with high risk of developing metabolic and cardiovascular disorders [47]. It stands with a good agreement with our results as we observed increased levels of leptin and decreased amount of adiponectin in adipocytes from EMS individuals. Previous studies demonstrated that PTP1B inhibition enhanced insulin sensitivity and adiponectin serum levels in monkeys [48]. Increased adiponectin concentration and expression following MSI-1436 may be explained by improved insulin sensitivity in adipocytes. Furthermore, activation of PPARγ activity positively regulates adiponectin secretion [49]. Therefore, it is likely that PTP1B inhibition results in the activation of PPARγ activity, which further triggers the cascade of events leading to increased adiponectin secretion and enhanced insulin sensitivity. It is supported by the recent findings by Kumar et al. [50], who revealed that Src homology region 2 domain-containing phosphatase-1 (Shp1)—the member of protein-tyrosine phosphates family—interacts with PPARγ by tyrosine phosphorylation. In case of leptin, it was shown that PTP1B deletion increases leptin sensitivity [51]. Here, we showed that MSI-1436 significantly reduces secretion of leptin by EMS derived adipocytes, which may protect against development of insulin resistance and restore physiological adipocyte secretome. It stands with a good agreement with Lund et al. [52] who revealed that negative regulatory role of PTP1B on leptin signaling is mediated through dephosphorylation of JAK2 and STAT3. Our findings revealed that PTP1B inhibition promotes adipogenesis which is in line with the research performed by Owen et al. [46] who showed enhanced lipogenesis in adipocyte-specific-PTP1B knockout mice. To summarize that part of research, it should be stated that PTP1B acts as a critical regulator of adipogenesis promoting progenitor stem cell differentiation. Yet, the results of further experiments clearly highlight additional signaling pathways that are under PTP1B influence in these cells.

Dimerization of insulin receptor followed by the insulin receptor substrate phosphorylation triggers two major signaling pathways—Ras to mitogen-activated kinases (MAPK) and phosphatidylinositol 3 kinase (PI3K) pathway, which elicits AKT/PKB kinase phosphorylation [53]. AKT is phosphorylated and activated by PDK1/2, and further phosphorylates AS160. As a consequence, GLUT4 translocation to membrane and glucose uptake is triggered. Interestingly, in the presented study, we observed increased expression of both isoforms *Akt1* and *Akt2* in EMS-derived cell/s in both ND and AD conditions. However, the strong limitation is lack of their protein levels, which may not correlate with mRNA levels. Inhibition of PTP1B resulted in the decreased expression of *Akt1* and *Akt2*. On the contrary, it was shown that PTP1B deficiency enhances AKT activation however in macrophages [54]. Due to relatively low data regarding PTP1B influence on *Akt* expression, that phenomenon requires further investigation. In the presented study, we also investigated the expression of two novel biomarkers of metabolic disorders—fetuin-A and SHBG. It was shown that fetuin-A levels are increased in adipose tissue of obese subjects [55], which correlates with our results as elevated expression of that gene was observed in EMS-derived cell/s. Contrary phenomenon was observed for SHBG, in which low serum levels correlate with increased susceptibility of type 2 diabetes development [15]. Yet, PTP1B inhibition did not alter expression of these genes, which indicates that PTP1B is not directly involved in the modulation of their action and works through distinct mechanisms.

Our previous, extensive research clearly reported on the increased senescence and apoptosis of EMS derived ASC [20, 22, 33]. In this study, we have shown that EMS cells in ND condition are characterized by increased expression of pro-apoptotic genes, including *p53*, *Bax*, and *p21*. It correlates with the studies performed by our and other research groups which showed that aging, lifestyle, and health condition strongly affect cytophysiological properties of mesenchymal stem cells [18, 21, 56–59]. Interestingly, we observed increased apoptosis in healthy cells in AD and EMS cells treated with MSI-1436 during differentiation. It can be explained by recent research performed by Hou et al. [60], who revealed that apoptosis is essential for early adipogenesis and that loss of caspase 3 impaired the differentiation capacity of preadipocytes. That phenomenon is explained by the hypothesis in which apoptotic cells stand as a source of factors and nutrients which facilitate adipogenic differentiation. It correlates with our findings which showed reduced caspase 3 in EMS derived ASC suffering from adipogenesis impairment. On the other hand, restoration of caspase 3 activity after MSI-1436 application enhanced adipogenic differentiation of EMS cells. Interestingly, it also enhanced the activity of caspase 9, which indicates that it can also be implicated in the differentiation process. Obtained results reveal a unique role of PTP1B as a modulator of apoptosis in progenitor stem cells triggered by adipogenic differentiation stimuli. This novel mechanism may stand as a potential therapeutic target in the development of strategies for adipose tissue metabolism modulation during insulin resistance.

In the next step of the experiment, we decide to investigate the expression of ER stress-related genes as EMS cells are characterized by UPR activation [22]. Furthermore, it was also shown that progressive ER stress inhibits adipocyte differentiation [61]. It correlates with our results as increased expression of *Chop*, *Perk*, *Atf6*, *Ire1*, and *Xbp1* was observed in EMS cells. However, inhibition of PTP1B significantly reduced the expression of the majority of these genes. Han et al. [61] demonstrated that IF2α-CHOP axis suppressed adipogenesis and limits expansion of fat mass in mice. On the other hand, Panzhinskiy et al. [62] reported that ER stress upregulates expression of PTP1B and impairs glucose uptake in cultured myotubes, while Agouni and colleagues [63] reported that PTP1B inhibition reduces ER stress-induced apoptosis in endothelial cells. For that reason, we can conclude that PTP1B inhibition results in increased adipogenic differentiation through the amelioration of ER stress.

Oxidative stress has been implicated in the development of multiple disorders including insulin resistance and type 2 diabetes [64]. EMS-derived cell/s were characterized by increased ROS and nitric oxide accumulation while MSI-1436 reversed that phenomenon. That fact explains observed here increased adipogenic differentiation after PTP1B silencing as it was shown that ER stress inhibits adipogenesis by the modulation of mitochondrial dynamics [65]. Interestingly, EMS cells cultured in ND condition displayed increased expression of *Sod1* and *Sod2* while their expression was diminished after MSCI 1436 application. We hypothesize that it can be a compensatory mechanism triggered by cells in order to deal with excessive accumulation of ROS. Yet, a strong limitation is a lack of *Sod1* and *Sod2* protein levels which may differ from their mRNA amount cells. Given the implication of oxidative stress in the onset of diabetes, increased antioxidative defense of cells treated with PTP1B inhibitor indicates on its potential utility against development of insulin resistance in obese subjects.

Finally, using GC-MS, we analyzed fatty acid composition in investigated cells. Free fatty acids (FFAs) are abundant in obesity leading to impairment of cellular metabolism through mitochondrial damage [66]. Interestingly, EMS differentiated cells exhibited lower amounts of palmitic acid (C16:0) and stearic acid (C18:0), and higher abundance of palmitoleic acid (C16:1) and linoleic acid (C18:3N, 6Z, 9Z, 12Z) as compared to healthy cells. Interestingly, high levels of palmitoleate are associated with the development of metabolic disorders [67]. Similar phenomenon was observed for plasma concentration of linoleic acid [68–70]. It was shown that oleic acid regulates adipogenesis through DNA methylation and may predispose to obesity and obesity-related disorders [71]. On the other hand, palmitate was shown to trigger adipogenic differentiation in bone marrow mesenchymal stem cells, which would explain that its insufficient amount in EMS cells may contribute to adipogenesis impairment as well [72]. To summarize, FFA composition strongly affects the adipocyte phenotype and may induce insulin resistance as well as deteriorate adipokine secretion profile. Here, we have shown for the first time that PTP1B inhibition affects composition of FFA in differentiated adipocytes which indicates on the link between PTP1B and lipid metabolism.

## Conclusions

Involvement of adipose tissue in the development of insulin resistance, obesity, and type 2 diabetes has been intensively investigated during the recent years. Searching for novel pharmaceuticals targeting that tissue brings hope to affected individuals. Selective chemicals, targeting molecules directly involved with the deterioration of adipocyte metabolism, may restore proper functionality of these cells and restore adipose tissue homeostasis. In the presented research, we tested MSI-1436—a selective PTP1B inhibitor on metabolically impaired progenitor cells—and obtained data revealed that it can restore adipogenic potential of these cells. Here, we have shown that ASC derived from EMS individuals suffers from adipogenesis impairment; however, PTP1B inhibition restores their plasticity and differentiation potential. Obtained results clearly indicate that inhibition of PTP1B modulates adipogenesis and lipid accumulation and highlights the therapeutic potential of its inhibitors against metabolic disorders. Here, we demonstrated that PTP1B acts as a negative regulator of progenitor stem cells plasticity which impairs adipogenic differentiation during insulin-resistant state. Our study provides an evidence of PTP1B role in metabolic disorders and shows that its inhibition during that state remodels adipose tissue plasticity and fatty acid composition restoring efficient differentiation of progenitor stem cells.

## Data Availability

The datasets generated during and/or analyzed during the current study are available from the corresponding author on reasonable request.

## Abbreviations

7-AAD: 7-Aminoactinomycin D
AD: Cells which underwent adipogenic differentiation
ASCs: Adipose stem progenitor cells
AT: Adipose tissue
ATF-6: Activating transcription factor
BrdU: Bromodeoxyuridine
DAPI: 4′,6-Diamidino-2-phenylindole
DIO: Diet-induced obesity
DMEM: Dulbecco’s modified Eagle’s medium
EI: Electron impact
EMS: Equine metabolic syndrome
ER: Endoplasmic reticulum
FFA: Free fatty acid
GC-MS: Gas chromatography-mass spectrometry
gDNA: Genomic DNA
HBSS: Hanks’ Balanced Salt Solution
HRP: Streptavidin horseradish-peroxidase
IKK: IκB-α kinase
IL-1: Interleukin-1
IRE-1: Inositol-requiring enzyme
JNK: c-Jun NH2-terminal kinase
ND: Undifferentiated cells
PERK: PKR-like ER-regulating kinase
PS: Penicillin and streptomycin
PTP1B: Protein tyrosine phosphate 1B
PTPs: Protein tyrosine phosphates
ROS: Reactive oxygen species
RT-PCR: Real-time reverse transcription polymerase chain reaction
SHBG: Sex hormone binding globulin
TNF-α: Tumor necrosis factor α
UPR: Unfolded protein response
